# 17β-Estradiol Enhances the Response of Plasmacytoid Dendritic Cell to CpG

**DOI:** 10.1371/journal.pone.0008412

**Published:** 2009-12-23

**Authors:** Xiaoxi Li, Yixin Xu, Ling Ma, Lingyun Sun, Gengfeng Fu, Yayi Hou

**Affiliations:** 1 Immunology and Reproductive Biology Lab, Medical School and State Key Laboratory of Pharmaceutical Biotechnology, Nanjing University, Nanjing, Jiangsu, People's Republic of China; 2 Drum Tower Hospital, Nanjing University Medical School, Nanjing, Jiangsu, People's Republic of China; 3 Jiangsu Centers for Diseases Prevention and Control, Jiangsu, People's Republic of China; 4 Jiangsu Key Laboratory of Molecular Medicine, Nanjing, Jiangsu, People's Republic of China; University of Miami, United States of America

## Abstract

Gender differences in immune capabilities suggest that sex hormones such as estrogens were involved in the regulation of the immunocompetence. Numerous studies also suggest that plasmacytoid dendritic cells (PDCs) play a pathogenic role in SLE. However, it is unclear whether estrogen can modulate the function of PDCs to influence the development of SLE. In the present study, PDCs from murine spleens were treated with 17β-estradiol (E2) and CpG respectively or both in vitro, then cell viability, costimulatory molecule expression, cytokine secretion of PDCs, as well as stimulatory capacity of PDCs to B cells were analyzed. Results showed that E2 and CpG increased the cell viability and costimulatory molecule expression on PDCs synergistically. Moreover, the intracellular and extracellular secretion of IFN-α was increased by E2 or E2 plus CpG. In addition, E2 and CpG also increased the stimulatory capacity of PDCs to B cells, and the viability of B cells was decreased after neutralizing IFN-α significantly. In the experiments in vivo, mice received daily s.c. injections of E2 and CpG respectively or both, then we found that the plasma concentration of IgM were elevated by E2 and CpG synergistically and the expression of IFN-α/β in spleens were noticeably increased by CpG plus E2 compared with the treatment of E2 or CpG only. This study indicates that E2 could exacerbate PDCs' activation with CpG, which further activates B cells to upregulate susceptibility to autoantigens. IFN-α plays an important role in the stimulatory effect of PDCs on B cells. E2 stimulation of IFN-α production may result in female prevalence in autoimmune diseases such as SLE through activation of PDCs. This study provides novel evidence of relationship between estrogen and SLE and also sheds light on gender biases among SLE patients.

## Introduction

The female prevalence in autoimmune diseases has been recognized for over 100 years. Evidence from murine models also showed the difference in basic immune responses between male and female [1]. Some reports suggested that systemic lupus erythematosus (SLE) patients experience an increase in flares during pregnancy, possibly due to the sustained increased levels of estrogen [2]–[4]. Lupus precipitated or exacerbated after commencement of oral contraceptive use [5]. Lahita and Bradlow reported that patients with SLE and their first-degree relatives had elevated serum levels of 16 -hydroxyestrone, an actively femianizing metabolite of 17β-estradiol (E2) [6], [7]. Moreover, Pisetsky et al reported that female mice displayed high levels of circulating DNA [8]. Our previous study showed that E2 could increase lymphocyte apoptosis [9]. It has proved that the average length of DNA separated from the anti-DNA antibody immune complex of SLE blood was 180 bp, in accordance with the size of apoptosis chromatin [10]. So scientists and physicians have suspected that steroid hormones such as estrogen may be a key regulator of autoimmune diseases including SLE [11].

Human and mouse plasmacytoid dendritic cells (PDCs) can recognize CpG representing a unique microbial molecule, leading to their activation and maturation [12], [13]. PDCs have been shown to correspond to a specialized cell population that produces large amounts of type I interferons such as IFN-α [14]. The production of the type I IFN by PDC in response to CpG is mediated through TLR9 which is specific for CpG-containing motifs [15]. On the other hand, numerous studies have suggested that PDCs play a pathogenic role in SLE. The infiltration of PDCs was found in the inflammatory site of SLE [16]. Recent studies have shown that DNA-containing complexes within SLE serum stimulate PDCs to produce IFN-α [17], [18]. Many SLE patients have increased serum levels of type I IFNs [19], [20]. IFN-α levels also correlate with anti-double-stranded DNA antibody production, complement activation which are important indicators in SLE disease progression [21]. In addition, a 23-year-old woman with a metastatic carcinoma developed SLE syndrome during IFN-α therapy [22]. These data suggested that PDC may be participated in the development of SLE through changing IFN-α secreting.

B cell dysfunction leading to a general B cell hypoactivity is also a characteristic of SLE [23], [24]. B cells have been thought to contribute to lupus through the production of autoantibodies such as anti-dsDNA antibodies [25], [26]. Moreover, PDCs and CpG license human B cells for plasma cell differentiation in the absence of T-cell help [27]. In vitro the virus-specific antibody production from B cells could be completely abolished by depleting PDCs from total blood mononuclear cells [28], influenza virus-induced type I interferon lead to polyclonal B-cell activation [29]. All these above suggest that B cells activation and differentiation may be mainly dependent on PDCs, but the pathways of their interaction need to be further studied.

Our previous study showed that E2 modulated the maturation and stimulatory functions of myeloid dendritic cells from healthy and SLE mice [30], [31]. However, it is unclear whether E2 can influence the recognition of PDCs on CpG and the interaction between PDCs and B cells. Therefore, in this study the response of PDC to CpG and the stimulatory functions of PDCs to B cells were investigated under the presence of E2 to understand the role of estrogen in SLE.

## Materials and Methods

### Animals

Female BALB/c and C57B6 mice, 6–8 weeks old, were bought from Model Animal Research Center of Nanjing University. MRL/lpr mice in different weeks old were obtained from Shanghai SLAC Laboratory Animal Co. LTD. They were bred and kept under standard-pathogen-free conditions in the animal facilities of the affiliated Drum Tower Hospital of Nanjing University Medical School (Nanjing, China). Mice were maintained for at least 1 week prior to use. All animal work was approved by the Animal Care Committee at Nanjing University.

### Regents

17β-estradiol, Hepes and CFSE were purchased from Sigma Chemical Company (St. Louis, MO, USA). IMDM and Phenol-free RPMI 1640 were bought from Gibco. The charcoal-stripped FCS was purchased from HyClone Laboratories (Logan, UT, USA). FITC anti-mouse CD40, CD80, CD86, B220, IFN-α and IgG2b, and PE anti-mouse CD19 were bought from eBioscience Inc (San Diego, CA, USA). Cell Counting Kit 8 was bought from Dojindo Laboratories (Japan). Mouse IFN-α EIISA Kit was bought from PBL Biomedical Laboratories (NJ, USA). Mouse IgM ELISA Quantitation Kit was bought from BETHYL (TX, USA). IFN-α/β antibody for immunohistochemistry analysis were purchased from Bioworld Company. Rat anti-mouse Interferon Alpha antibody for neutralizing mouse interferon alpha was purchased from PBL Biomedical Laboratories. Estradiol ELISA Kit (Cat. No. 10–4310) was purchased from DSL Laboratories, INC. Mouse Plasmacytoid Dendritic cell Isolation Kit, B cell Isolation Kit and anti-Ly-6C-APC, anti-mPDCA-1-FITC were purchased from Miltenyi Biotec (Bergisch Gladbach, Germany). Dead Cell Discriminator (DCD) was obtained from Caltag Laboratories. E2 was dissolved at 10 mM in water-free ethanol, then diluted in RPMI 1640 medium to a concentration of 10 µM stored at −20°C until use. The CpG with the sequence 5′-TCC ATG ACG TTC CTG ACG TT-3′ was synthesized from Shanghai SBS Genetech Technology Co., LTD. CpG were dissolved in Tris-EDTA buffer and used at a final concentration of 3 µM.

### Single-Cell Preparation from Mouse Spleen

Mice were immediately sacrificed and spleens were immediately removed and placed in small culture dishes containing sterile PBS with 10% FBS. The splenocytes were dissociated by gently pressing the organ through a fine, sterile 30 µm-nylon mesh. The mesh and culture dish were rinsed with medium, and then cell suspension was collected in sterile 15 ml conical tubes. Erythrocytes were removed by treating the splenic cells with ACK lysing buffer (0.15 M NH_4_Cl, 1.0 mM KHCO, 0.1 mM EDTA, pH 7.2) for 4–5 min and washing twice with PBS.

### Isolation of PDCs and B cells

For isolation of PDCs, single-cell suspension prepared from total spleen was incubated for 20 min at 4°C with Biotin-Antibody Cocktail, FcR Blocking Reagent and Anti-Biotin MicroBeads (Miltenyi). Then the labeled cells were depleted with LS column in order to obtain the purified PDCs. PDCs were cultured in phenol-free RPMI-1640 supplemented with 10% FCS, 100 U ml^−1^ penicillin, 100 µg ml^−1^ streptomycin, 2 mM L-glutamine, 10 mM Hepes and 1 mM sodium pyruvate. Similar to the isolation of PDCs for B cells, single-cell suspension was incubated for 20 min at 4°C with Biotin-Antibody Cocktail (CD43, CD4 and Ter119) and Anti-Biotin MicroBeads, and then the labeled cells were depleted with LS column in order to obtain the B cells fraction. The purity of PDCs and B cells were determined by fluorescently stained with anti-Ly-6C-APC, anti-mPDCA-1-FITC and anti-CD19-PE (Figure S1 and Figure S2). The cell debris and dead cells were excluded from the analysis based on scatter signals and DCD fluorescence. Then all the PDCs were divided into four groups: Control, E2 (10^−8^ M), CpG (3 µM) and Both (10^−8^ M E2+3 µM CpG).

### Mice Ovariectomy and E2/CpG Exposure

Thirty BALB/cJ mice were randomly divided into six groups: Control (n = 10), sham+vehicle (n = 10), Ovx+vehicle (n = 10), Ovx+E2 (n = 10), Ovx+CpG (n = 10), Ovx+E2+CpG (n = 10). Mice were surgically ovariectomized (n = 40) or sham-operated (n = 10) after anesthesia using sodium pentobarbital. All animals were well owed to recover for two weeks and then received daily s. c. injections of either E2 (100 µg/kg/day) or CpG (1.2 mg/Kg/day) only or both which were dissolved in sesame oil or PBS every morning for 2 weeks. Mice were euthanized 24 h after receiving their last injection.

### Plasma Collection and Measurement of E2 Concentration

Mice were immediately sacrificed and cardiac blood was collected from each group or MRL mice and centrifuged at 3500 rpm. Plasma was obtained and stored at −70°C until use. Plasma was then used to verify E2 concentration using a commercial Estradiol EIA Kit according to the manufacturer's instruction. Briefly, plasma samples or E2 standards were added to the 96-well micro titer plates coated with mouse monoclonal antibody. Specific antibody to E2 and Tracer (acetylcholinesterase linked to E2) were added. The plate was then covered and incubated for 1 h at room temperature. The wells were washed five times and added Ellman's reagent for 60–90 min at room temperature with gentle shaking. The plate was read at a wave length of 412 nm.

### Measurement of Immunoglobulins in the Plasma

The level of IgM and IgG in plasma was detected by mouse IgM/IgG ELISA Quantitation kit (Bethyl Laboratories, Montgom, TX) according to the step by step protocol.

### Cells Viability Assay

The cell number of every group was adjusted to 2×10^5^ ml^−1^ and seeded into round-bottom 96-well microculture plates for 100 µl with six parallel wells for each group. After they were cultured for 72 h, the PDCs or B cells in the wells were assayed for cell viability by using a Cell Counting Kit-8 according to the manufacturer's instructions. First, cell suspension (100 µl/well) was inoculated in a 96-well plate, and the plate was pre-incubated in a humidified incubator at 37°C, 5% CO_2_. Then 10 µl of the CCK-8 solution was added to each well of the plate, and incubated for 6 h in the incubator. At last, the cell viability was measured at the absorbance 450 nm of reduced WST-8(2-(2-methoxy-4-nitrophenyl)-3-(4-nitrophenyl)-5-(2,4-disulfophenyl)-2H-tetrazolium, monosodium salt) using a microplate reader (Bio-Tek).

### Cells Proliferation Assay

PDCs or B cells (in MLR assay) at 4×10^6^ cells/ml were stained with 2 µM CFSE in pre-warmed PBS for 10 min at 37°C, washed in medium twice, incubated in pre-warmed medium for another 5 min, and washed again. The cells then were adjusted to 1×10^6^ cells/ml and cultured in medium with accordance treatment as described above and incubated for 72 h. After 72 h cells were harvested and washed in PBS/1% BSA/0.05% sodium azide and analyzed by flow cytometry with logarithmic detection of a green fluorescence (CFSE). Events were gated to exclude the cell aggregate.

### Phenotype Assay by Flow Cytometry

Purified PDCs treated with E2 and/or CpG ODN for 24 h were washed twice in FACS medium phosphate buffered PBS containing 1% FCS and 0.1% NaN3. Then the cells were incubated for 30 min at 4°C with FITC-labeled CD40, CD80 and CD86 antibody according to the standard procedure, and followed by fixation in 1% paraformaldehyde. Isotype controls were used for each antibody. Fluorescence was measured using a FACScan flow cytometry (Becton Dickinson) and data analysis was performed using the Cell Quest Software (Becton Dickinson, San Diego, CA).

### Intracellular Cytokine Analysis by Flowcytometry

The PDCs treated with E2 and/or CpG for 24 h in the presence of 1.7 µg/ml monensin were washed twice in PBS containing 1% FCS and 0.1% NaN_3_, and then fixed in 2% formaldehyde and permeabilized with 0.5% saponin for 20 min at 4°C respectively. Samples were washed with wash buffer containing 0.5% saponin and then incubated with FITC labeled IFN-α mAb for 1 h, then cells were washed with 0.5% saponin three times and resuspended in 300 µl of 1% paraformaldehyde in PBS. Finally, PDCs were analyzed using Cellquest Software (Becton Dickinson, San Diego, CA, USA) as the described above.

### Enzyme-Linked Immunosorbent Assay of IFN-α

ELISA kits of IFN-α were conducted according to the manufacture's protocol (RD). Briefly, every group of cells was cultured for 24 h. Then the cell culture supernatant or IFN-α standards were added to the 96-well micro titer plates coated with mouse monoclonal antibody in triplicate or duplicate, and incubated for 1 h in a closed chamber at 24°C. Following washing once, the antibody solution was added to each well and incubated for 24 h at 24°C. Afterwards, the well was washed three times, HRP Conjugate solution added to each well, and incubated for 1 h at 24°C. Following washed four times, TMB substrate solution was added to each well and incubated for 15 min at 24°C in the dark. Then the stop solution was added to each well. At last, the absorbance was determined at 450 nm using a microplate reader (Bio-Tek).

### Preparation and Staining of Frozen Tissue Sections

The freshly isolated tissue samples including spleen and kidney were cut into the desired number of 0.5 cm^3^ pieces and then were made into the OCT embedding compounds. The specimens were incubated 5–10 minutes in liquid nitrogen and then stored at −80°C for long time storage. The OCT-embedded tissues were cut to 5 µm thick sections and fixed in acetone for 5 min, then left the slides to air dry 10 min at room temperature. After the fix step, slides were treated with 0.3% H_2_O_2_/PBS for 5 min or until bubbling stops in order to eliminate endogenous peroxidase activity, and 5% standard goat serum was added to the slides for 15 min blocking. Primary antibody of IFN-α/β was diluted in blocking buffer at the ratio of 1∶100 and incubated for one night at 4°C in the humidified chamber. After washing in the PBS, sections were incubated with the HRP labeled goat anti-rabbit second antibody (1∶50, Beyotime Institute of Biotechnology, cn.) for 30 min at 37°C in the humidified chamber. The DAB chromogenic reagent kit (Wu Han Boster, Biological Technology.) was used to colorate at room temperature for 1–30 minutes. The reaction was stopped by washing with distilled water. At last, the slides were counterstained with hematoxylin for 50 sec, washed 10 min in tap water, dehydrated through a graded series of ethanol and transparent with xylene until sealed slides.

### Allogeneic Mixed Lymphocyte Reaction (MLR In Vitro)

Functional activity of PDCs was reflected in the primary allogeneic MLR assay. B cells as responder cells were obtained from mouse spleen by using B cell isolation kit (Miltenyi). The MLR assays were carried out in 96-well round-bottom microplates to ensure efficient PDC/B cell contact. pDCs were treated with E2 and/or CpG for 24 h and then B cells were implanted with PDC at the ratio of 1∶10 (2×10^4^∶ 2×10^5^ per well) for 72 h in CO2 incubator (the aim of designing one ratio was that the stimulatory capacity of this ratio is high and that it can fully reflect the influence of E2 and CpG on this capacity of PDCs). Meanwhile, in another parallel assay, 1 ng/ml neutralizing antibody to IFN-α was added to the coculture system after PDCs 24 h treating, and then cell viability and proliferation were assayed by using the CCK-8 and CFSE separately.

### Statistical Analysis

Each experiment was performed three or four times, data were expressed as mean ±standard deviation (SD). Statistical analyses were performed with Graphpad Prism 5.0 by using the repeated measures ANOVA and the ANOVA post Bonferronites test. Differences were considered statistical significant when P<0.05.

## Results

### E2 Influences the Cell Viability of PDCs

**Figure 1:**
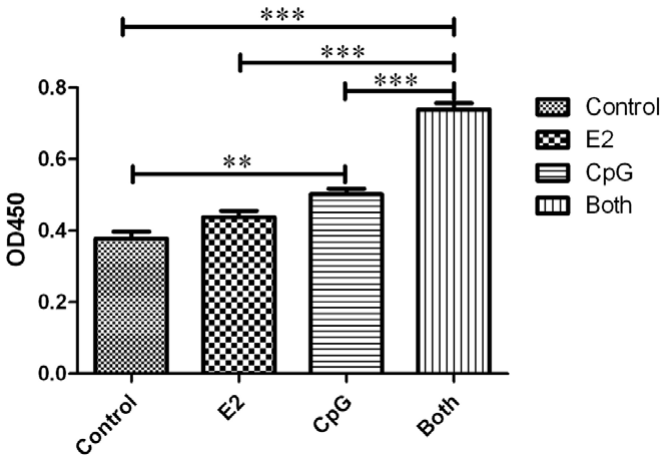
The viability of PDCs. The cell viability was determined by Cell Counting Kit. The results are presented as mean ± S.D. with triplicate measurement. Comparisons between the different stimuli are indicated by *, ** and ***: p<.05, .01 and .005, respectively; n = 6.

Activation of PDCs improves their cell counts in culture and upregulates their expressing co-stimulatory molecules [32]. We isolated the PDCs from the spleen of Balb/c mice and cultured with E2, CpG respectively or both for 72 h. Our results showed that the viability of PDCs treated with CpG or CpG+E2 were increased especially in the group under the presence of E2 at the same time. E2 had a moderate effect of PDCs without CpG but had no statistical significance (Figure 1). In addition, we used the CFSE assay to see if the PDCs had proliferation under such treatments, and data (Figure S3) reflected no proliferation of PDCs. These phenomena above indicated that CpG and E2 influence the viability of PDCs.

### E2 Enhances the Expression of Co-Stimulatory Molecules CD40 and CD86 on PDCs Exposed to CpG

**Figure 2:**
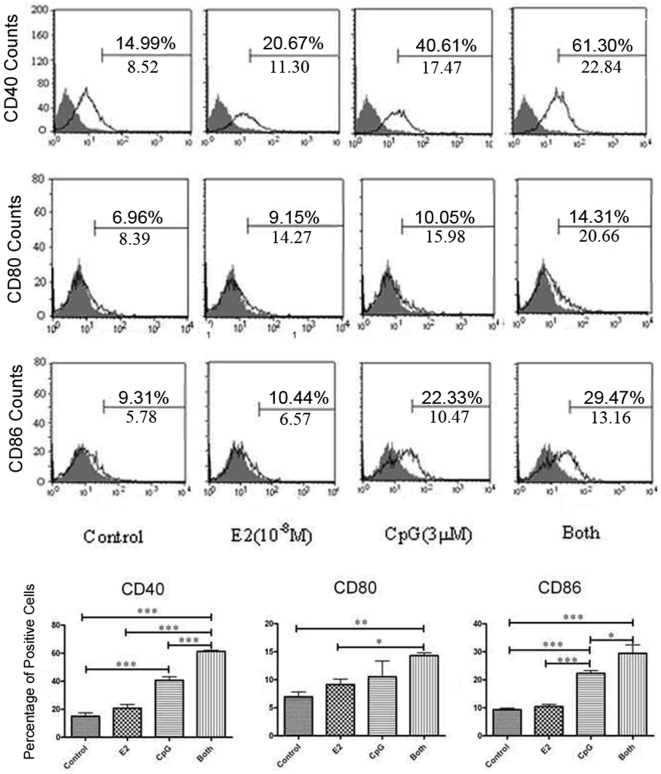
The expression of co-stimulatory molecules on PDCs. The PDCs were gated to fall within established viable cell forward and side scatter parameters and to eliminate the cell aggregates. The surface expression of costimulatory molecules CD80, CD86 and CD40 on purified pDCs under the treatment with E2 and/or CpG in vitro were further analyzed in a FL1 density plot. Data are reported as positive PDC cells and MFI. Histograms shown are representative of three experiments with homogenous results. Comparisons between the different stimuli are indicated by *, ** and ***: p<.05, .01 and .005, respectively; n = 3.

In order to determine the activation and maturation of the PDCs, we detected the co-stimulatory molecules expression on them. The results showed that CpG promoted the expression of CD40 and CD86 after 24 h. CD80 expression was also detected but the change was not very obviously. At the same time, E2 alone influenced little on co-stimulatory. However, when combination of both E2 and CpG was used, the enhancements of CD40 and CD86 expression were more strikingly than CpG alone (Figure 2 and Figure S4). The results suggested that E2 could enhance the PDCs' activation and maturation by changing the CD40 and CD86 expression.

### E2 Elevates the Intracellular and Extracellular Level of IFN-α Produced by PDCs with the Stimulation of CpG

**Figure 3:**
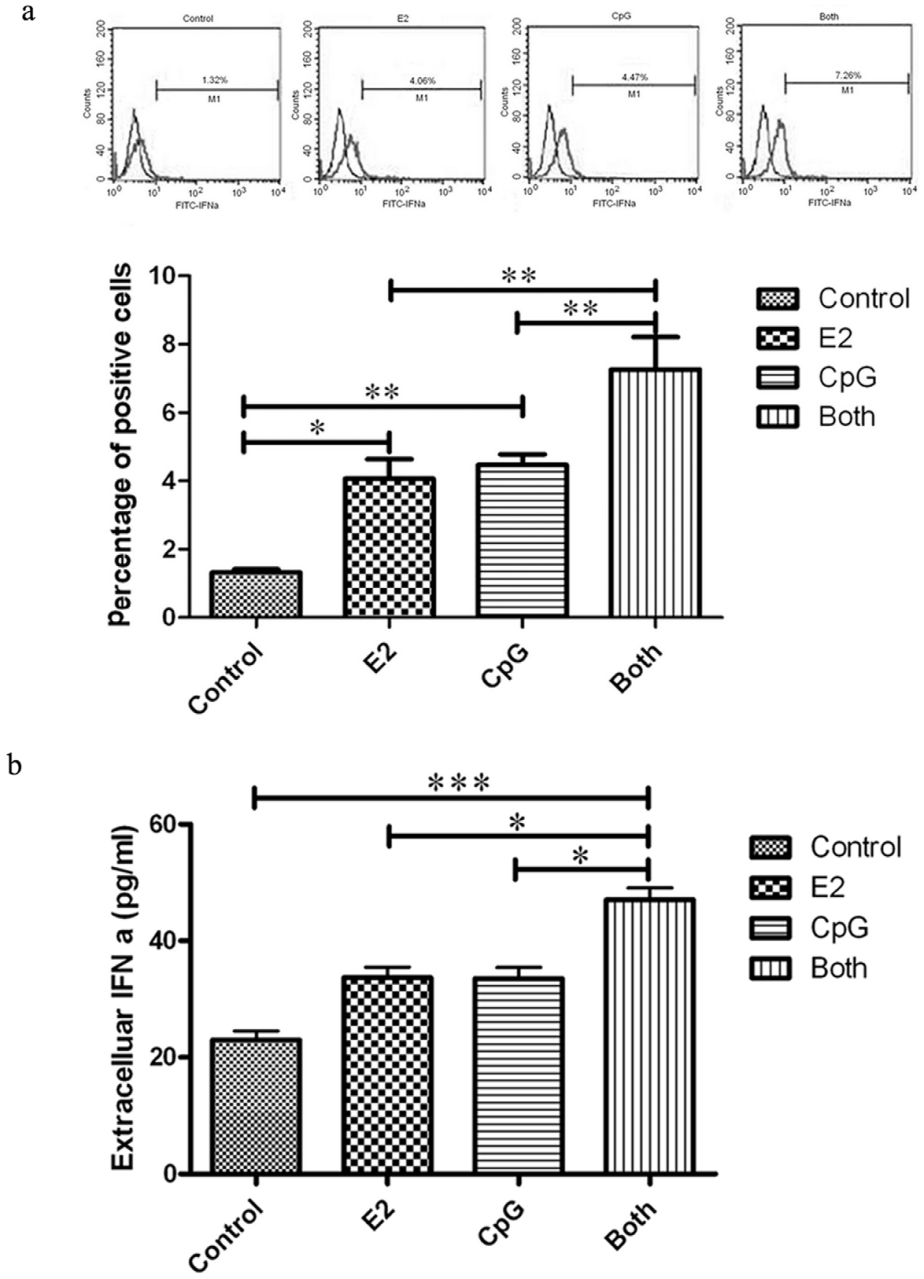
a The intracellular expression of IFN-α. The intracellular IFN-α expression in PDCs was analyzed by FCM, cells were gated to fall within established viable cell forward and side scatter parameters and to eliminate the cell aggregates. Histograms shown are representative of three experiments with homogenous results. Comparisons between the different stimuli are indicated by *, ** and ***: p<.05, .01 and .005, respectively; n = 3. **b The secretion level of IFN-α.** PDCs from the four groups were treated with E2/CpG separately or E2 plus CpG for 24 hours. Then the cell culture was collected and assayed using the IFN-α ELISA Kit. The absorbance at 450 nm was read using a microplate reader (Bio-Tek). The results are presented as mean ± S.D. with triplicate measurement. Comparisons between the different stimuli are indicated by *, ** and ***: p<.05, .01 and .005, respectively; n = 3

PDCs are professional type I IFN-producing cells upon viral infection [33]. PDCs in SLE patients appear to be constantly activated through TLR9 by self-chromatin-antichromatin antibody complexes to produce type I IFNs [34]. In order to research the manner of which E2 and/or CpG regulate PDCs' secretion of IFN-α, we tested both intracellular and extracellular levels of IFN-α after PDC was cultured for 24 h in the presence of E2 and/or CpG (Figure 3a and Figure 3b). Results showed that E2 or CpG could increase the level of IFN-α, while E2 plus CpG elevated the level of IFN-α more significantly than that of E2 or CpG alone. This suggests that E2 could enhance PDCs to express both intracellular and extracellular IFN-α levels when the cells were activated by CpG.

### E2 Improves the Stimulatory Capacity of PDCs on B Cells with the Activation of CpG through IFN-α

**Figure 4:**
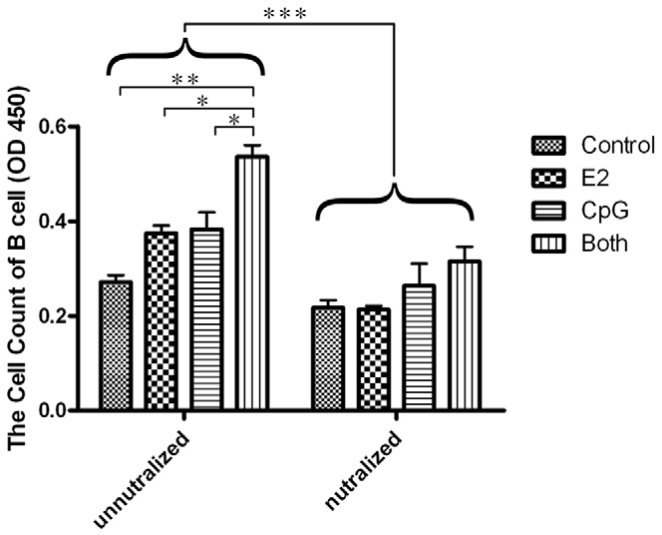
The viability of B cells in MLR and the effect of IFN-α in MLR. After treated with E2 and/or CpG 24 h, 1 ng/ml neutralizing antibodies for IFN-α were added or not added to the culture system, PDCs mixed with B cells in ratio of 1∶10 for 72 h. Then cell proliferation was determined by Cell Counting Kit. The results are presented as mean ± S.D. with triplicate measurement. Comparisons between the different stimuli are indicated by *, ** and ***: p<.05, .01 and .005, respectively; n = 3.

B cells activation and differentiation is dependent on PDCs [27]–[29]. We designed MLR assays to test whether E2 and/or CpG could enhance the effect of PDCs' stimulatory capacity on B cells. The results of CCK-8 assay showed that after co-culture with the treated PDCs for 72 h, the viability of B cells in E2 or CpG group was increased compared to that in control group, while the viability was more notable in both group than in either E2 or CpG group, and a statistical significance was observed when compared with CpG group only (Figure 4). In the CSFE assay, we did not observe obvious change of MFI between the different experimental groups (Figure S5). So we concluded that the data reflected B cells viability but not proliferation. Then, we used anti-mouse IFN-α antibody to neutralize the IFN-α in the culture supernatant, the viability of B cells was all significantly decreased in E2, CpG and Both groups (Figure 4). This data indicated that either E2 or CpG could increase the stimulatory capacity of PDCs to thereby enhance B cells' viability. Moreover, E2 enhances the effect of PDCs on B cells in combination with CpG, and IFN-α may play a key role in the regulation of PDC function. We also offer the pictures of MLR with or without neutralizing for IFN-α in order to show the states of cell growth. The colors of cell culture supernatant were different because of the different states of cell growth (Figure S6 and Figure S7).

### Plasma Estradiol Levels

**Figure 5:**
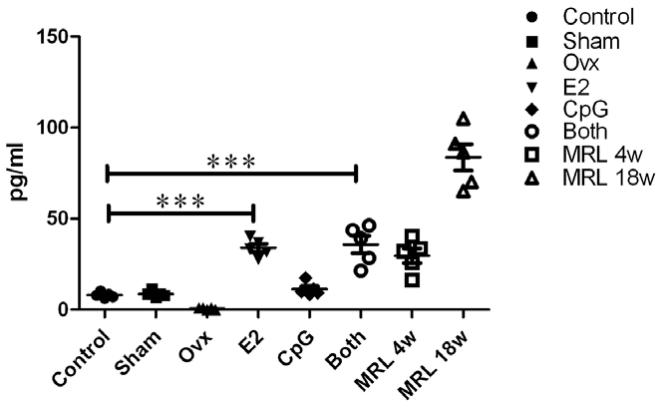
The level of E2 in the plasma. After two weeks treatment with E2 and CpG separately or together, the plasma from the different groups was collected and tested using the Estradiol ELISA Kit. The plate was read at a wave length of 412 nm. The results are presented as mean ± S.D. Comparisons between the different stimuli are indicated by *, ** and ***: p<.05, .01 and .005, respectively; n = 5.

Estradiol EIA Kit was checked and the inter-assay coefficient of variation (CV) in ELISA was 2.77%. The results detected by ELISA confirmed that plasma E2 concentration in Ovx+E2 group mice was more than 4 times as much as that in virgin (control) mice, and almost the same as that in 4 weeks old MRL mice, and nearly the half of that in 18 weeks old MRL mice. Plasma E2 concentrations were 8.03±1.53 pg/ml (control, n = 10), 8.61±1.67 pg/ml (sham+vehicle, n = 10), 34.02±4.78 pg/ml (Ovx+E2 n = 10), 9.28±1.46 pg/ml (Ovx+CpG, n = 10), 35.83±10.52 pg/ml (Ovx+E2+CpG, n = 10), 30.03±12.43 pg/ml (4 w MRL), and 87.25±17.43 pg/ml (18 w MRL), respectively (Figure 5). E2 could not be detected in Ovx+vehicle treated group. The normal physiological range of plasma E2 concentration in the mouse was reported to be between 5 and 50 pg/ml [35].

### Plasma IgM Levels

**Figure 6:**
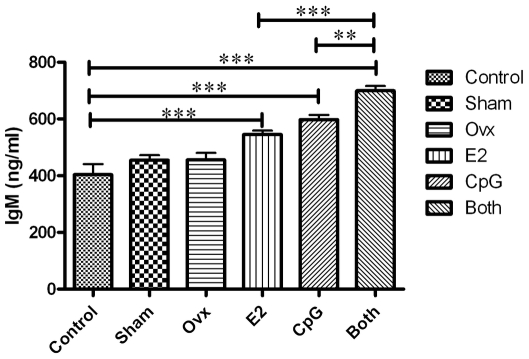
The plasma concentration of IgM. The IgM concentration was checked by Estradiol EIA Kit according to the manufacturer's instruction. The absorbance at 450 nm was read using a microplate reader (Bio-Tek). The results are presented as mean ± S.D. Comparisons between the different stimuli are indicated by *, ** and ***: p<.05, .01 and .005, respectively; n = 3.

In order to further understand the interaction between PDCs and B cells, we measured the immunoglobulins produced by B cells. IgG was detected very low in all the groups, and IgM's secreting was obviously increased by E2, CpG and E2 plus CpG (Figure 6). Compared with E2 or CpG only, E2 plus CpG synergistically enhanced the IgM level in the mice plasma. This result had a same trend as the change of E2 level in vivo and the IFN-α secreting of PDCs.

### E2 and CpG Increased the IFN-α Expression in Mouse Spleen but Not Kidney

**Figure 7:**
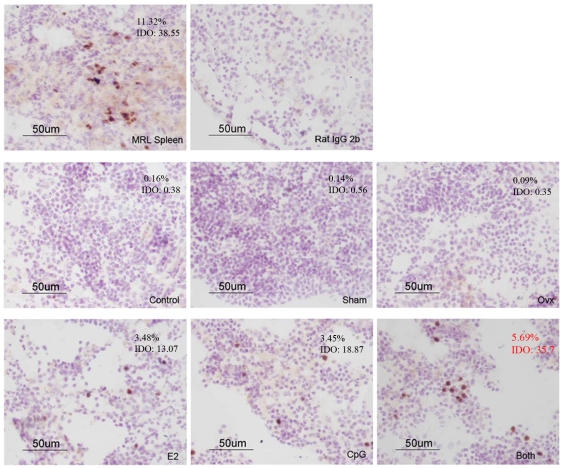
The expression of IFN-α/β in the spleens of mice. Immunohistochemical staining was used to detect the expression of IFN-α/β in the spleens from mouse treated with E2 and/or CpG. The average of the percentage of IFN-α^+^ cells is from more than 5 mice from each group under 40× magnification.

**Figure 8:**
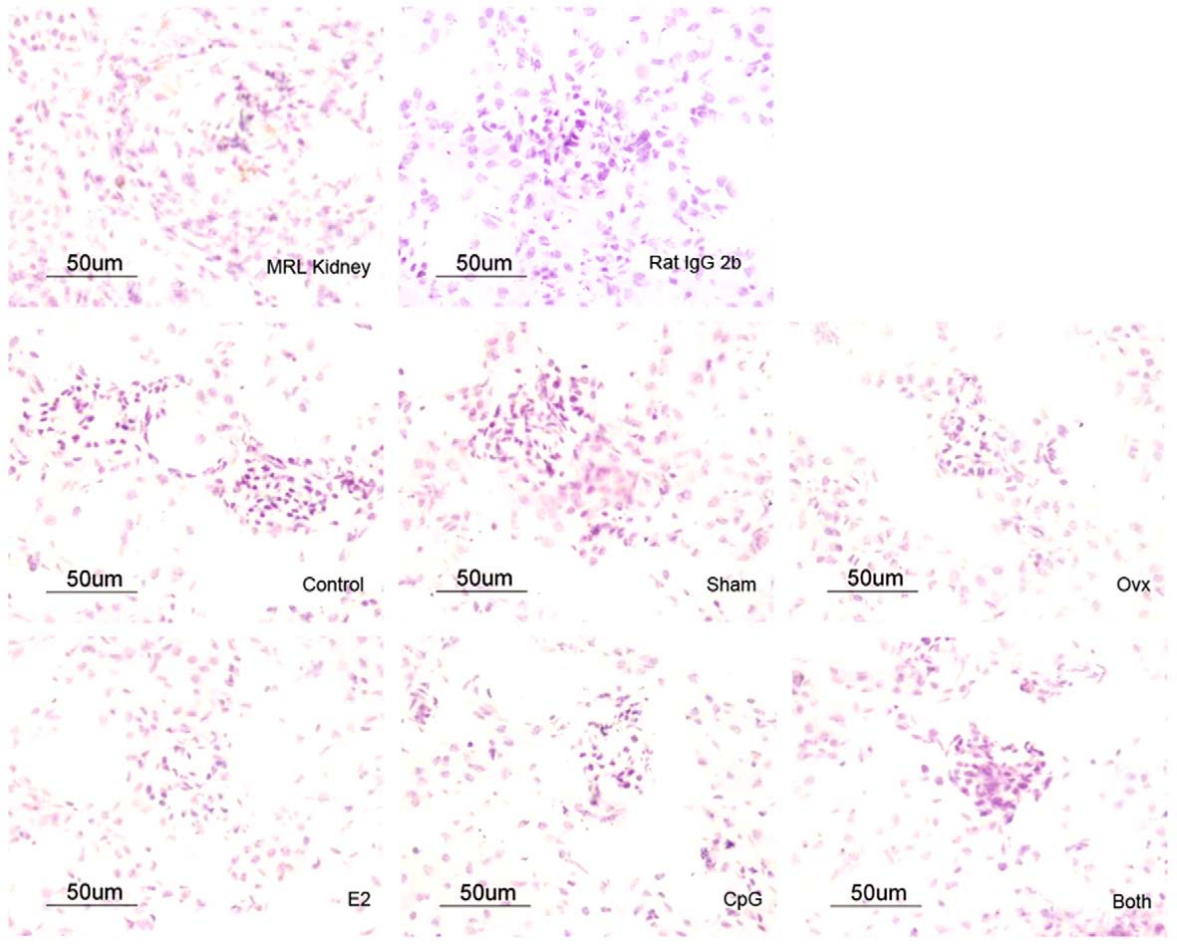
The expression of IFN-α/β in the kidney of mice. Immunohistochemical staining was used to detect the expression of IFN-α/β in the kidneys from mouse treated with E2 and/or CpG. The average of the percentage of IFN-α^+^ cells is from more than 5 mice from each group under 40× magnification.

Since the IFN-α played a so important role in the E2 and CpG regulation of PDCs in vitro, it is necessary to determine whether IFN-α could expression on the spleen or kidney tissues with the treatment of E2 and/or CpG. Meanwhile, to further understand the biological role of type I IFN in the background of estrogen related diseases, such as SLE, we checked the IFN-α/β expression on the tissues from each group, and compared them with the MRL mice tissues. The percentage and IDO (integrated optical density) of IFN-α+ cells were analyzed using the software Image-Pro 6.0. The results showed that the IFN-α/β expression in the spleen of group Both were further increased compared with that in group E2 and CpG, and that's similar to the spleen from MRL mice. In addition, the expression of IFN-α/β was undetected in the kidney of every group. (Figure 7 and Figure 8)

## Discussion

E2 is believed to etiology of both human and murine SLE, but there is little knowledge about the immune state of PDCs under the E2 environment. Recent studies have identified PDC as the central cell type which could secret a large amount of IFN-α upon CpG motifs, and the purified PDC but not mDC represents the primary target for CpG. CpG increased survival, activation and maturation of PDCs [36]. So the purified PDCs from murine spleens were used for our studies in vitro to explain the higher prevalence of SLE in females.

In order to understand the effects of CpG and E2 on the activation of PDCs, we firstly test the viability and the expression of co-stimulatory molecules on PDCs. Our results showed that E2 plus CpG could significantly increase the viability of PDCs than CpG or E2 only. Meanwhile, the co-stimulatory molecules CD40 and CD86 were also upregulated by E2 and CpG separately or together, but the effect of E2 on CD80 expression was minimal compared with CD40 and CD86. These results was consistent with the report from Anne Krug that CpG could promote survival of PDCs, activate purified PDCs by a rapid increase of CD80, CD86 and CD40 expression but the increased degree of CD80 was much lower than that of CD86 [37]. Amaya lparraguirre reported that although both influenza virus and CpG led to increased expression levels of costimulatory molecules on PDCs, including CD40 and CD86 as well as MHC II, an exception was CD80, which was expressed to the lower extend under the CpG stimuli than the influenza virus stimuli [38]. This suggested us that the costimulatory expression may be depended on different ligands. Compared with CD11c^+^ DCs, since the level of expression of these molecules remained always lower, this seemed to suggest that PDCs may be not good at stimulating effector cells in adaptive immunology, and E2 may be involved in modulating the function of PDCs with exposure to CpG.

PDC is thought to express TLR9 that is specialized to detect intracellular pathogens such as viruses and intracellular bacteria or parasites based on CpG motifs within their DNA. PDCs are also the major producers of IFN-α, which plays an important role in the clearance of viruses and other intracellular pathogens, and have the ability to produce 100–1000 times more type I IFN than the other blood cell types following virus activation [39]. Recent data indicated that DNA-anti-DNA complexes in SLE sera can provide an activation signal for IFN-a secretion [40]. It was also shown that apoptotic cells induce PDCs to produce IFN-a [41], [42]. Because of the DNA, in bacterial, virus or immune complexes can be mimicked by synthetic CpG motifs [43], we designed the experiment to test if E2 and CpG could aggregate PDCs activation respectively or both through detecting the IFN-α secreting. Our results showed that E2 plus CpG could significantly increase the level of IFN-α compared with E2 or CpG alone. These data suggest that PDCs are dedicated to produce much more IFN-α in anti-nucleic acid autoimmunity under the presence of E2. So we presume that E2 inflicts PDCs to product gross IFN-α with the stimulation of CpG. Honda and Taniguchi reported that type I IFN induction was mediated trough TLR signaling, which stimulated sequential activation of adaptors and kinases of the IRAK, IKK and TRAF families, and then leading to the activation of IRF5 and IRF7 [44], [45]. These related researches suggested us E2 maybe change the expression of IRF5 or IRF7 through MAPK signaling pathway so as to enhance the responses of PDCs to CpG, and eventually change the IFN-α secreting level, we will make further studies on the possible pathways of the interaction between E2 and CpG in our next research step.

IFN-α was found to potently enhance humoral immunity [46] and a number of studies highlighted an important role for DCs of the myeloid lineage in regulating B-cell differentiation by soluble factors [47] and via cell to cell contact [48]. So we further studied the interaction of PDCs with B cells. Our results showed that either E2 or CpG could increase the stimulatory capacity of PDCs to thereby enhance B cells' viability, and E2 enhanced the effect of PDCs when coupled with CpG. Anne Krug found that PDCs synergistically enhance activation and cytokine production of human peripheral blood B cells that were stimulated by B-cell receptor ligation (anti-Ig) and a microbial molecular (CpG) [36]. PDC–derived soluble factors including IFN-α contributed to B-cell activation and increased immunoglobulin production (IgM and IgG) of blood B cells [49]. Together with the above result that E2 and/or CpG could increase PDCs' IFN-α secretion, we propose that E2 activates B cells viability through promoting PDCs to secrete more IFN-α and keeping cell-to-cell contact state. So we used anti-mouse IFN-α mAb to neutralize the IFN-α that existed in culture system. The result showed that the viability of B cells was decreased after neutralizing the cytokine compared with the groups without neutralizing IFN-α. This suggests that IFN-α is necessary in regulating the interaction between PDCs and B cells under the treatment of E2 with CpG.

In order to further understand the effect of E2 on PDCs and B cells, we designed in vivo experiment that E2 and/or CpG were injected to different groups of mice, and then the E2 concentration and immunoglobulin production (IgM and IgG) of plasma, and IFN-α/β expression on tissues spleen and kidney were checked. The results showed that the E2 levels of the groups Ovx+E2 and Ovx+E2+CpG were similar to the 4 w MRL mice and nearly 4 times more than the Control and Sham groups which in line with our expectations. There were much more IFN-α/β expression in mouse spleen in the Both group than either E2 or CpG group, but IFN-α/β expression was undetected in the Control, Sham and Ovx group. In addition, IFN-α/β expression was also undetected in kidney. This is suggests that E2 may promote the migration of PDCs into spleen tissue so as to lead to the elevated IFN-α/β expression in the local region, and then the production of IgM was increased in the plasma. It is presumed that E2 could change B cells' activation in exposure to CpG or bacterial or immune complexes peptides in vivo. However, it was reported that PDCs numbers in blood cells of SLE patients are decreased [36], it seems contradictory with the high level of IFN-α in the sera of SLE. But it seems important for the local concentration of IFN-α in an affected region. According to our results IFN-α/β expressed higher in mouse spleen in the Both group than either E2 or CpG group, we proposed that PDCs may be recruited to lymphoid organs or inflamed tissues under the stimuli of CpG motifs, and E2 aggravated the process of autoimmune diseases.

In conclusion, E2 and/or CpG increase the cell viability of PDCs, and upregulate the expression of CD80, CD86 and CD40 on PDCs. E2 plus CpG also increase IFN-α secretion. In addition, E2 increased the stimulatory capacity of PDCs on B cells with CpG. In vivo, E2 could lead to the increased IFN-α/β expression in mouse spleens and exacerbate PDCs' activation, which further activated B cells to upregulate their susceptibility in producing autoaitigens. E2 may participate in the pathogenesis of the female prevalence in autoimmune diseases such as SLE through the activation of PDCs.

## Supporting Information

Figure S1 The purity of isolated PDCs. The purity of PDCs was determined by fluorescently stained with Anti-mPDCA-1-FITC and Anti-Ly-6C-APC. The cell debris and dead cells were excluded from the analysis based on scatter signals.
(3.71 MB TIF)

Figure S2 The purity of isolated B cells. The purity of B cells was determined by fluorescently stained with Anti-mCD19-PE. The cell debris and dead cells were excluded from the analysis based on scatter signals.
(3.32 MB TIF)

Figure S3 The proliferation assay of PDCs. PDCs were fluorescently stained with CSFE and then treated with E2 of CpG separately or together for 72 hours. The MFI of every group was analyzed by flow cytometry with logarithmic detection of a green fluorescence (CFSE). Events were gated to exclude the cell aggregate. Green cruve: Control group; Red curve: E2 group; Yellow curve: CpG group; Blue curve: Both Group.
(2.42 MB TIF)

Figure S4 The expression of co-stimulatory molecules on PDCs indicated by MFI. a) the MFI statistical chart of CD40; b) the MFI statistical chart of CD80; c) the MFI statistical chart of CD86. Histograms shown are representative of three experiments with homogenous results. Comparisons between the different stimuli are indicated by *, ** and ***: p<.05, .01 and .005, respectively; n = 3.
(5.63 MB TIF)

Figure S5 The proliferation assay of B cells in MLR. B cells were fluorescently stained with CSFE and then mixed with treated PDCs for 72 hours. The MFI of every group was analyzed by flow cytometry with logarithmic detection of a green fluorescence (CFSE). Events were gated to exclude the cell aggregate. Green cruve: Control group; Red curve: E2 group; Yellow curve: CpG group; Blue curve: Both Group.
(2.45 MB TIF)

Figure S6 The cell states from neutralizing group in the MLR test. The pictures of cells were photographed by Nikon optical microscope and enlarged 200× times. The colors of cell culture supernatant from each group were different because of the different states of cell growth.
(2.52 MB TIF)

Figure S7 The cell states from unneutralizing group in the MLR test. The pictures of cells were photographed by Nikon optical microscope and enlarged 200× times. The colors of cell culture supernatant from each group were different because of the different states of cell growth.
(2.51 MB TIF)
