# Supplementary figures and images for: 17β-Estradiol Enhances the Response of Plasmacytoid Dendritic Cell to CpG
Source: PLoS One. 2009 Dec 23;4(12):e8412. doi: 10.1371/journal.pone.0008412 (PMC2793013; doi:10.1371/journal.pone.0008412)

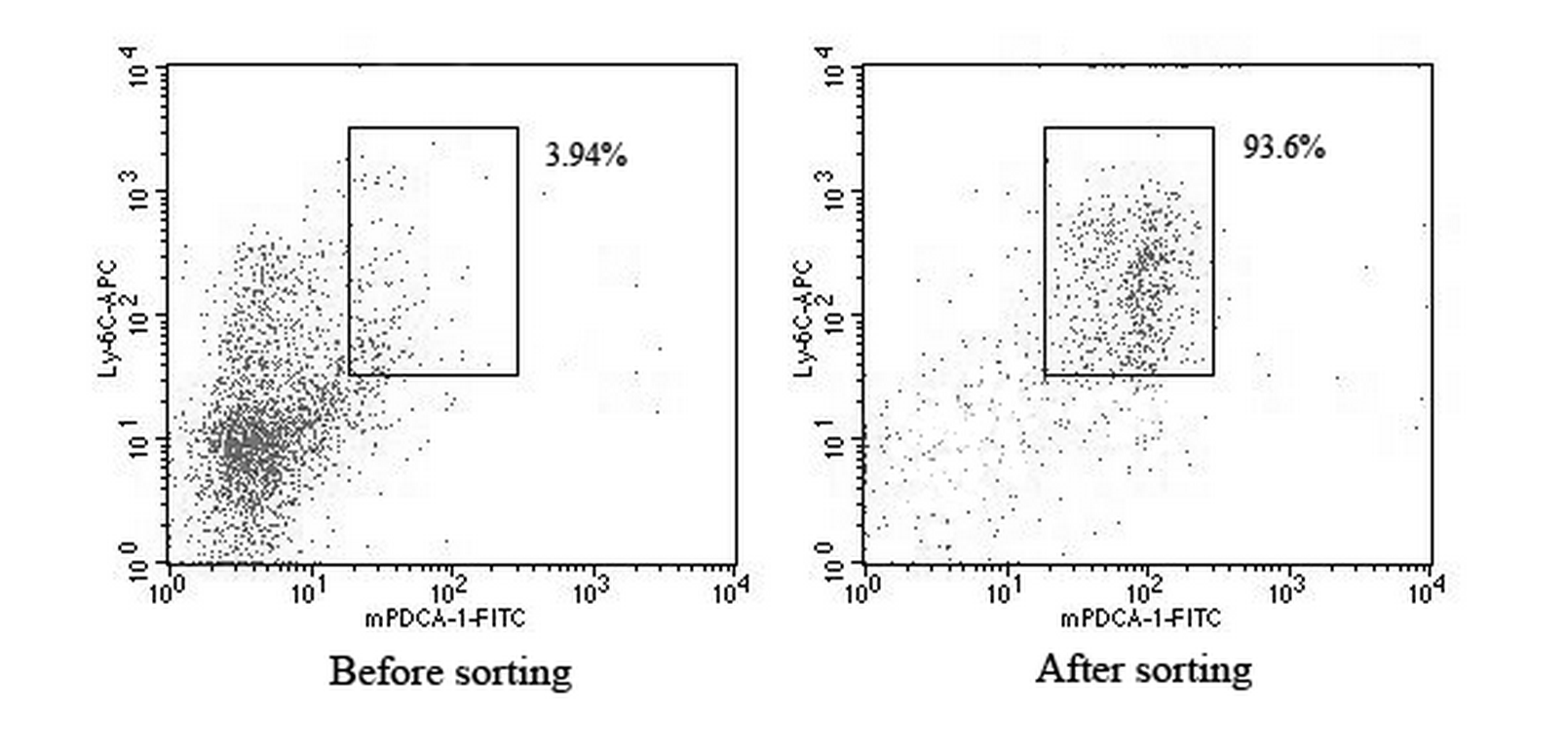

Supplement: Figure S1 — The purity of isolated PDCs. The purity of PDCs was determined by fluorescently stained with Anti-mPDCA-1-FITC and Anti-Ly-6C-APC. The cell debris and dead cells were excluded from the analysis based on scatter signals. (3.71 MB TIF) [file pone.0008412.s001.tif]

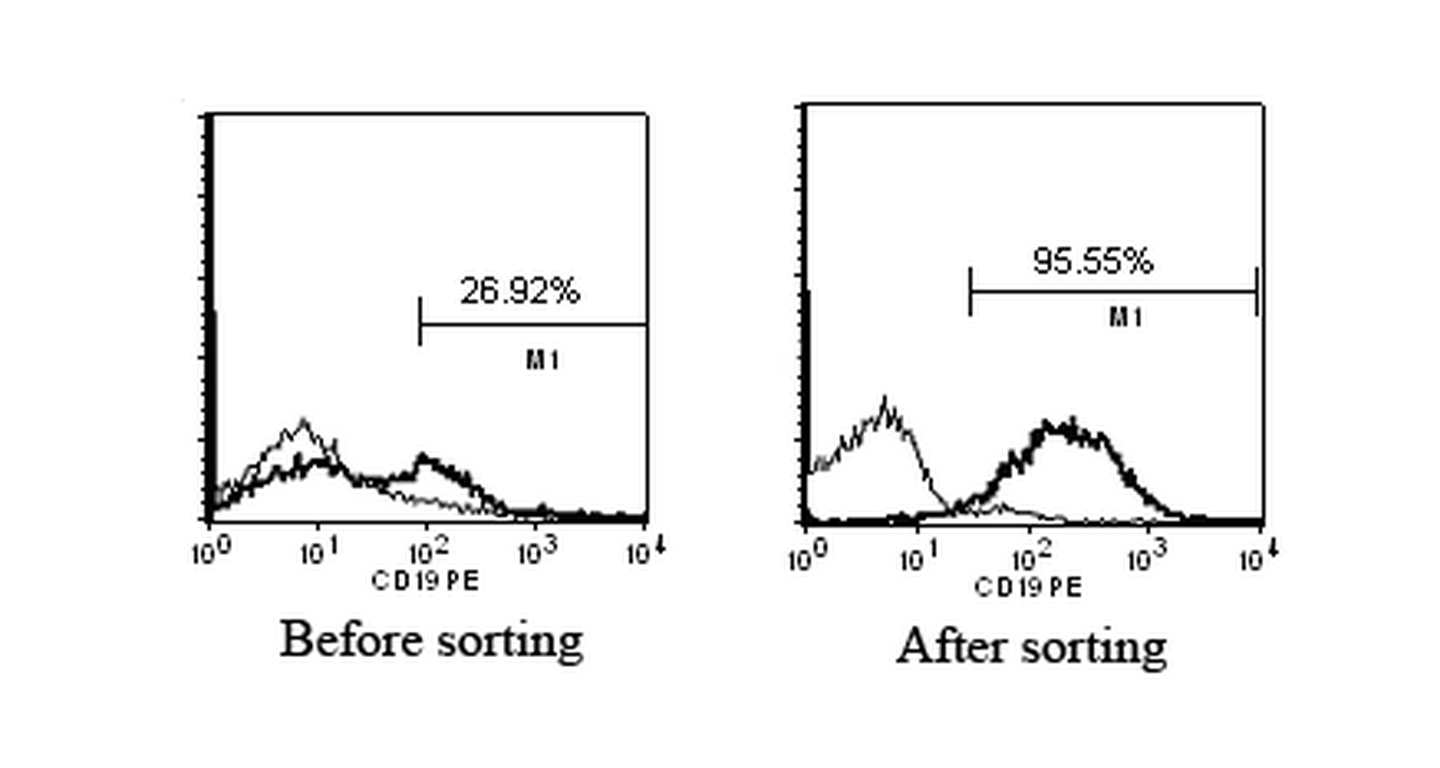

Supplement: Figure S2 — The purity of isolated B cells. The purity of B cells was determined by fluorescently stained with Anti-mCD19-PE. The cell debris and dead cells were excluded from the analysis based on scatter signals. (3.32 MB TIF) [file pone.0008412.s002.tif]

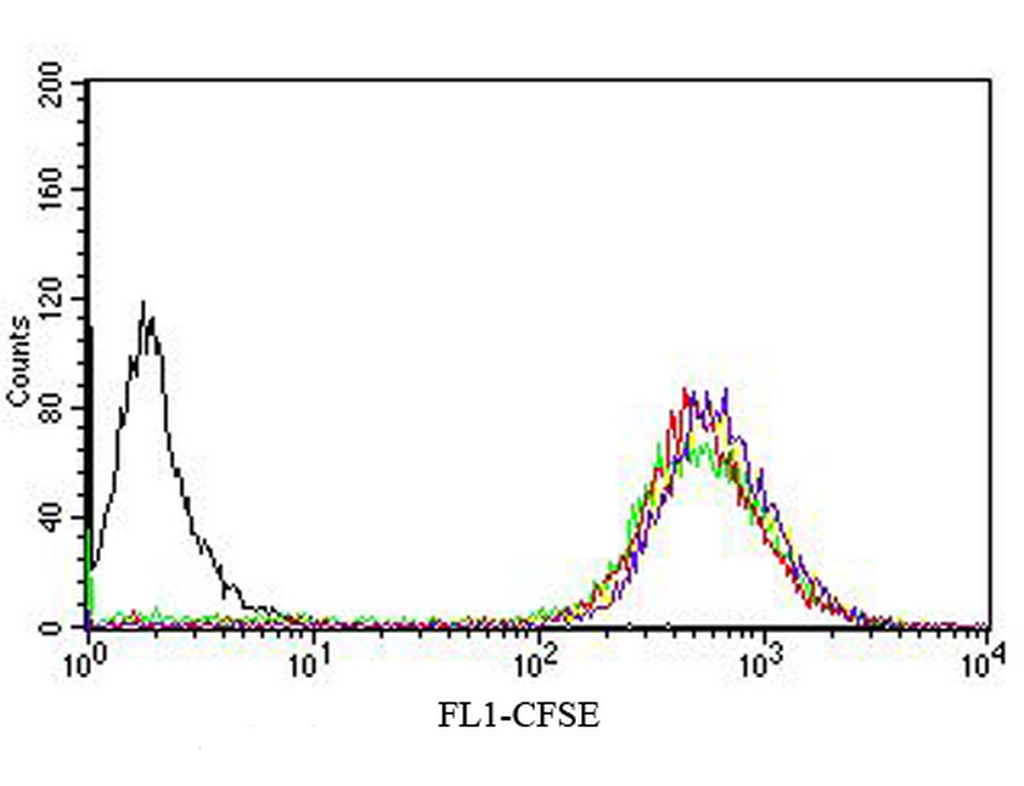

Supplement: Figure S3 — The proliferation assay of PDCs. PDCs were fluorescently stained with CSFE and then treated with E2 of CpG separately or together for 72 hours. The MFI of every group was analyzed by flow cytometry with logarithmic detection of a green fluorescence (CFSE). Events were gated to exclude the cell aggregate. Green cruve: Control group; Red curve: E2 group; Yellow curve: CpG group; Blue curve: Both Group. (2.42 MB TIF) [file pone.0008412.s003.tif]

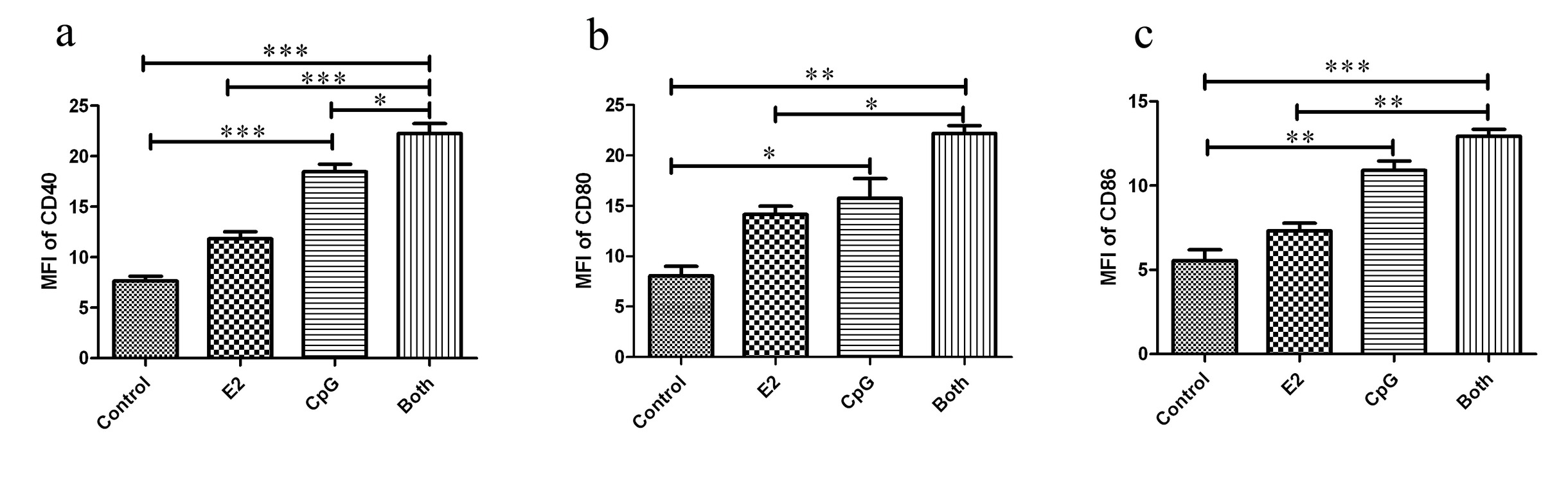

Supplement: Figure S4 — The expression of co-stimulatory molecules on PDCs indicated by MFI. a) the MFI statistical chart of CD40; b) the MFI statistical chart of CD80; c) the MFI statistical chart of CD86. Histograms shown are representative of three experiments with homogenous results. Comparisons between the different stimuli are indicated by *, ** and ***: p<.05, .01 and .005, respectively; n = 3. (5.63 MB TIF) [file pone.0008412.s004.tif]

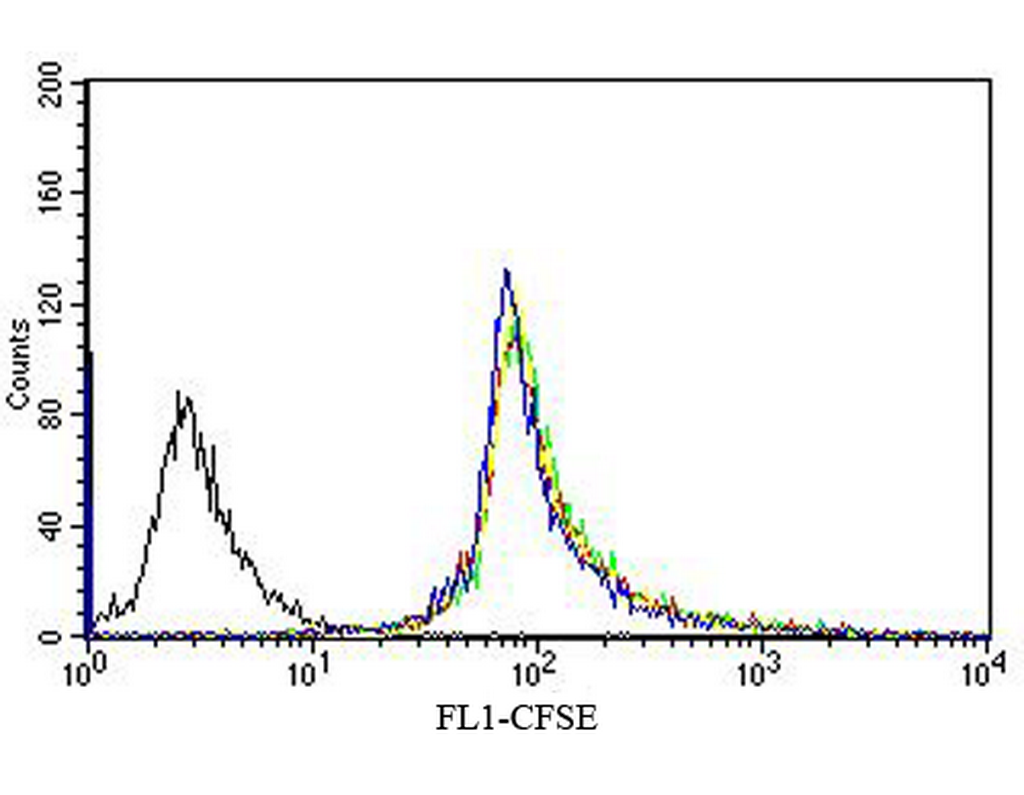

Supplement: Figure S5 — The proliferation assay of B cells in MLR. B cells were fluorescently stained with CSFE and then mixed with treated PDCs for 72 hours. The MFI of every group was analyzed by flow cytometry with logarithmic detection of a green fluorescence (CFSE). Events were gated to exclude the cell aggregate. Green cruve: Control group; Red curve: E2 group; Yellow curve: CpG group; Blue curve: Both Group. (2.45 MB TIF) [file pone.0008412.s005.tif]

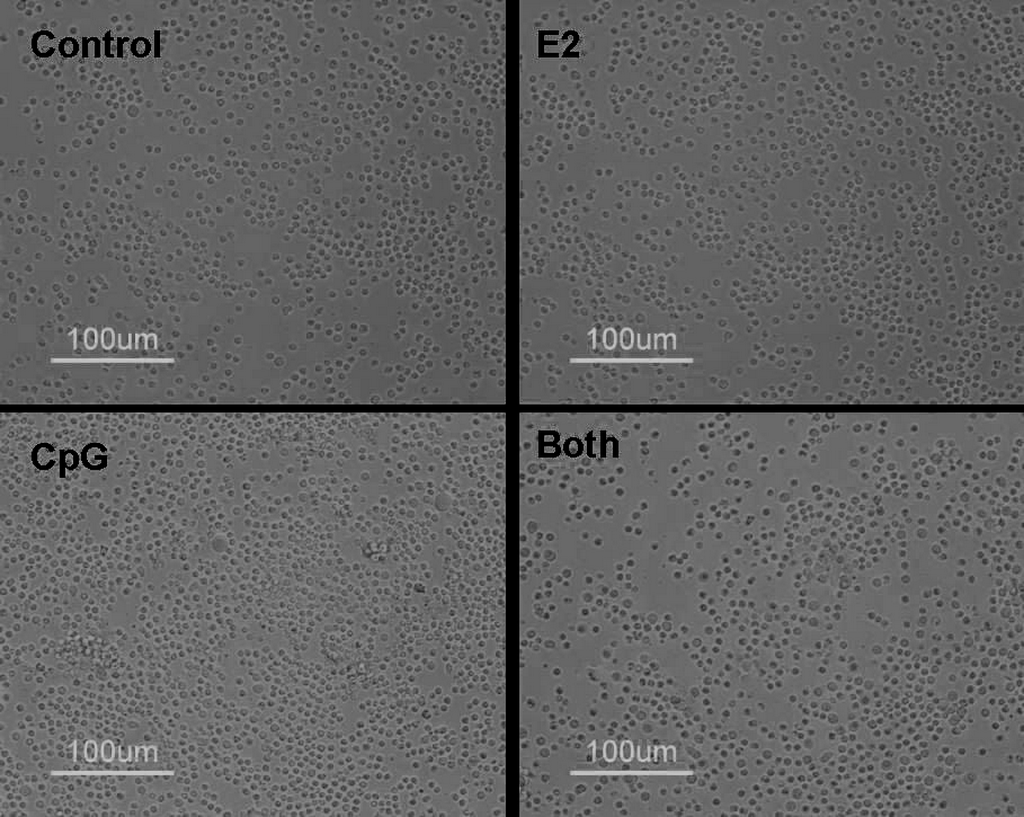

Supplement: Figure S6 — The cell states from neutralizing group in the MLR test. The pictures of cells were photographed by Nikon optical microscope and enlarged 200× times. The colors of cell culture supernatant from each group were different because of the different states of cell growth. (2.52 MB TIF) [file pone.0008412.s006.tif]

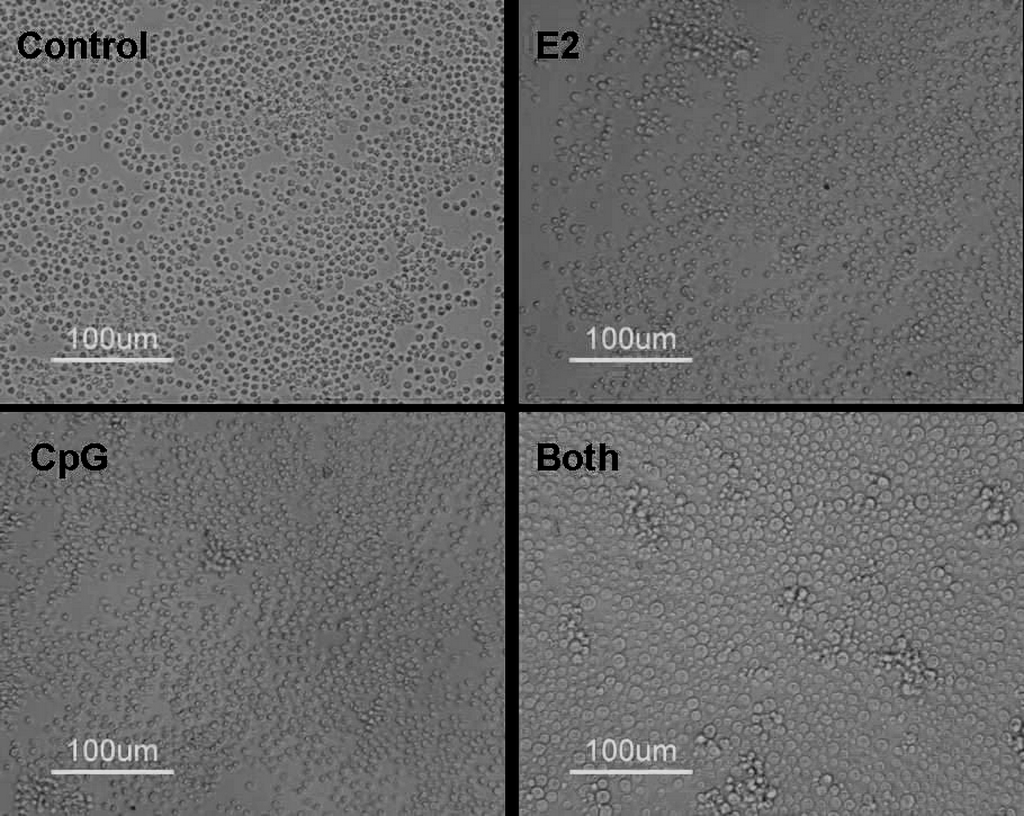

Supplement: Figure S7 — The cell states from unneutralizing group in the MLR test. The pictures of cells were photographed by Nikon optical microscope and enlarged 200× times. The colors of cell culture supernatant from each group were different because of the different states of cell growth. (2.51 MB TIF) [file pone.0008412.s007.tif]
